# Diversity of microbial communities in cigar filler leaves with different initial water contents analyzed based on high-throughput sequencing technology

**DOI:** 10.3389/fmicb.2025.1508866

**Published:** 2025-02-07

**Authors:** Yumeng Gao, Yihui Wang, Bingqing Hou, Guo Zhang, Chun Jiang, Song Fang, Qian Wang, Yang Ning

**Affiliations:** ^1^Tobacco Research Institute, Chinese Academy of Agricultural Sciences, Qingdao, China; ^2^Graduate School of Chinese Academy of Agricultural Sciences, Beijing, China; ^3^Shandong China Tobacco Industry Limited Company, Jinan, China; ^4^Anhui Wannan Tobacco Limited Company, Xuancheng, China

**Keywords:** cigar tobacco leaves fermentation, initial water content, microbial community, high-throughput sequencing, functional prediction

## Abstract

**Introduction:**

To study the composition and succession of bacterial and fungal communities during the fermentation of cigar filler leaves with varying initial water contents, high-throughput sequencing technology was used to sequence the bacterial 16SrRNA genes and fungal ITS1 genes from cigar tobacco leaf samples. This was followed by analyses of microbial *α*-diversity, microbial community structure, and bacterial function prediction based on the sequencing data.

**Results:**

The diversity and richness of microbial communities decreased over time during fermentation under different water content conditions. Among the 18 cigar filler leaf samples, the predominant phyla identified were *Proteobacteria*, *Firmicutes*, *Actinobacteria*, *Ascomycota*, and *Basidiomycota*, with the leading genera being *Staphylococcus, Sphingomonas, Methylobacterium-Methylorubrum*, *Pseudomonas*, and *Humicola.* Functional predictions for the bacteria revealed their primary involvement in carbohydrate, lipid, and amino acid metabolism.

**Conclusion:**

The initial water content of cigar tobacco leaves influenced the structure and relative abundance of microbial communities during fermentation. While the microbial community exhibited a similar structural composition, there were notable differences in relative abundance. The functional prediction results from PICRUSt indicated that the differences in predicted functional species among samples were minimal, whereas the variations in the abundance of functional species were more pronounced across different fermentation stages and initial water contents.

## 1 Introduction

Cigars are a unique tobacco product that necessitates high-quality raw tobacco leaves. A handmade cigar is made up of three components: the wrapper, the binder, and the filler. The filler is the central part of the cigar and significantly affects its flavor and aroma when smoked (Zhang et al., 2024). Fermentation is a crucial process in tobacco production (Hu et al., 2022; Toharisman et al., 2008), enhancing the color and sheen of the tobacco, softening the leaves, improving combustibility, and breaking down macromolecules like proteins and sugars into aroma precursors such as organic acids and carbonyl compounds, which enrich the flavor of cigar tobacco (Wu X. et al., 2023; Zhong et al., 2024; Rong et al., 2021; Di Giacomo et al., 2007), thereby greatly enhancing the smoking experience (Rodríguez-Bustamante et al., 2005).

The initial water content of tobacco leaves is a critical factor influencing the fermentation of cigar tobacco, as it determines the chemical transformations within the tobacco and subsequently affects its intrinsic quality (Song et al., 2023). An optimal initial moisture level can foster the growth of specific functional microorganisms during the pre-fermentation phase, leading to the accumulation of flavor-enhancing compounds. However, after air-curing, the water content of cigar tobacco leaves is low, which does not satisfy the moisture needs for microbial fermentation (Tian et al., 2018). Conversely, excessive moisture during fermentation can cause the tobacco leaves to become moldy (Pan et al., 2019). Research indicates that the water content of cigar tobacco leaves is also closely linked to changes in fermentation temperature: too much moisture can cause rapid heating of the leaves, resulting in uneven fermentation, while too little moisture leads to slow warming and also uneven fermentation (Qiao et al., 2018).

The researchers have shown that water content significantly influences the quality of fermentation, particularly in the solid-state fermentation of soybean curd pomace, where low water levels hinder fungal growth (Shi et al., 2012). Additionally, the Plackett-Burman test identified initial water content as one of the three key factors affecting the production of red yeast pigments through the solid fermentation of Rhizobium erythrorhizonticum (Ji et al., 2008). In the fermentation of soybean paste, the water content in the grains directly impacts microbial fermentation, which subsequently affects the aroma of the final product (Zhao et al., 2006).

Currently, high-throughput sequencing technology has been used for the screening and identification of functional microorganisms and enhancement of their roles during the air-curing stages and fermentation of cigar tobacco leaves (Zhang et al., 2023; Zhao et al., 2024; Walsh et al., 2023). However, there is limited research on how initial water content affects microbial community changes during the fermentation of cigar tobacco leaves. Therefore, this study aims to explore the shifts in microbial communities during the fermentation of cigar filler tobacco leaves from Anhui Province, utilizing Illumina Miseq high-throughput sequencing technology. This research will serve as a reference for future studies on the relationship between the development of cigarillo flavor and microorganisms in cigar filler tobacco leaves under varying initial water content conditions.

## 2 Materials and methods

### 2.1 Experimental materials

The experimental materials were sourced from the southern Anhui tobacco region in China (114°54′-119°37′E, 29°41′-34°38′N). This region is situated in a transitional zone between a subtropical monsoon humid climate and a warm-temperate monsoon semi-arid climate. It features a humid and mild climate, with moderate average rainfall that is evenly distributed. Over 80% of the soil in this area consists of sandy, sandy loam, or loamy sandy types, characterized by a loose texture and good drainage.

### 2.2 Sample collection

Samples of 5 kg of central cigar filler tobacco leaves were chosen and rehydrated to various target moisture levels (20, 22, 24%). They were then placed in self-sealing bags and put into a constant temperature and humidity oven, where the temperature was increased by 2°C each day until it reached 42°C. After closing the oven, the samples were allowed to cool to room temperature before being reheated, and this process was repeated multiple times. The fermentation was concluded based on the aroma of the tobacco and evaluations made. Each time the temperature reached the target level, 20 pieces of tobacco were collected and stored in a −60°C refrigerator. Each sample included three independent biological replicates. In the labeling of the cigar filler tobacco leaves samples, T1, T2, and T3 indicate different moisture levels, while 01–06 denote the six sampling instances throughout the fermentation process.

### 2.3 DNA extraction and sequencing

Using sterile scissors, 100 g of cigar filler leaves were taken separately into conical flasks. PBS buffer was added and allowed to thoroughly soak the tobacco leaves and shaken in a shaker at 37°C, 200 r/min, for 1 h. After shaking, the microorganism-containing PBS buffer was centrifuged for 5 min at 8,000 r/min in a centrifuge, and the precipitate was collected. Sample DNA was extracted using the E.Z.N.A. Soil DNA Kit (Omega Bio-tek, Inc., USA) kit. After extraction, the quality of the DNA was achieved through 1% agarose gel electrophoresis, and the concentration and purity were measured with a NanoDrop 2000 (Thermo Fisher Scientific Inc., USA). An appropriate amount of DNA extract was taken in a centrifuge tube and diluted to 1 ng/μL using sterile water.

### 2.4 PCR amplification

Following this, PCR amplification was conducted, and the details of the amplification region and sequence are provided in the table. PCR reaction parameters: pre-denaturation at 94°C for 5 min, denaturation at 94°C for 30 s, annealing at 55°C for 30 s, extension at 72°C for 1 min, 30 cycles, and final extension at 72°C for 7 min. The size of the amplified target bands was verified using 1% agarose gel electrophoresis. The PCR products were automatically purified with the Agencourt AMPure XP (Beckman Coulter, Inc., USA) Nucleic Acid Purification Kit, followed by library construction using the NEB Next Ultra DNA Library Prep Kit (New England Biolabs, Inc., USA). Sequencing was carried out on the Illumina MiSeq PE 300 platform by Beijing Ovison Gene Technology Co.

**Table:** 

Type	Region	Primer name	Primer sequence(5′ - 3′)
16S	V5-V7	799F	AACMGGATTAGATACCCKG
		1193R	ACGTCATCCCCACCTTCC
ITS	ITS1	ITS1F	CTTGGTCATTTAGAGGAAGTAA
		ITS12R	GCTGCGTTCTTCATCGATGC

### 2.5 Sequencing data processing

The sequencing data were filtered and assembled with Pear (v0.9.6) software. The resulting optimized sequences were categorized into Operational Taxonomic Units (OTUs) based on 97% similarity using Usearch software. The representative sequences of the OTUs were matched against the Unite 8.2 database (Abarenkov et al., 2010) using the BLAST algorithm (Ye et al., 2006), with an e-value threshold of 1e-5, to identify the species taxonomic information for each OTU. The *α*-diversity index was calculated from the OTUs and their abundance using QIIME (Caporaso et al., 2010) (v1.8.0) software. The community composition of species at each taxonomic level was statistically analyzed to determine the structure of the microbial community, and predictive analysis of bacterial function was conducted using PICRUSt.

### 2.6 Data analysis

The structural composition of the microbial community was obtained by statistically analyzing OTUs for abundance, α-diversity, and community results of species at each taxonomic level. Data was processed using SPSS 27 software and Microsoft Excel 2019, drawing with R (v3.6.0) software. R (v3.6.0) igraph and psych package were used for the network of interactions. Microbial data analysis was conducted on the Allwegene Cloud Platform, and visualizations were created using Origin 2021.

## 3 Results

### 3.1 High-throughput sequencing depth, OTUs, dilution curve analysis

High-throughput sequencing of 18 samples yielded a total of 1,365,616 sequencing pairs (Reads) for bacteria, which after double-ended Reads QC and splicing yielded a total of 871,871 Clean Reads, with each sample yielding at least 65,247, and a total of 1,470,816 pairs of Reads for fungi, which after double-ended Reads QC and splicing yielded 1,441,605. 1,470,816 pairs of Reads were obtained from the fungus, and 1,441,605 Clean Reads were generated after double-ended Reads quality control and splicing, producing at least 71,995 reads per sample (Table 1).

**Table 1:** Sequencing results of samples during fermentation of cigar filler leaves under different water content conditions.

Sample	Fermentation stage	Initial water content (%)	Original sequence		Valid sequence		OTU	
			Bacteria	Fungi	Bacteria	Fungi	Bacteria	Fungi
JX-T1-01	Initial stage	20	70,758	78,742	45,195	78,091	428	113
JX-T1-02			83,618	81,185	41,811	76,342	724	79
JX-T1-03	Middle stage		76,797	84,630	34,320	84,314	672	93
JX-T1-04			76,074	80,131	56,029	79,922	547	106
JX-T1-05	Late stage		74,910	82,669	42,317	82,036	389	99
JX-T1-06			77,942	87,184	37,773	86,987	111	58
JX-T2-01	Initial stage	22	78,643	79,483	45,056	75,803	546	93
JX-T2-02			83,224	81,050	53,297	80,126	483	82
JX-T2-03	Middle stage		76,880	79,668	34,847	78,569	718	102
JX-T2-04			74,979	81,016	49,684	80,812	295	107
JX-T2-05	Late stage		70,681	81,619	53,505	80,415	114	94
JX-T2-06			65,247	81,563	59,480	80,760	109	63
JX-T3-01	Initial stage	24	76,342	79,628	45,138	71,995	669	105
JX-T3-02			84,664	82,509	53,487	77,599	215	64
JX-T3-03	Middle stage		79,809	84,527	50,632	84,003	408	118
JX-T3-04			79,096	82,509	42,117	82,315	423	105
JX-T3-05	Late stage		70,659	81,318	68,973	80,837	52	93
JX-T3-06			65,293	81,385	58,210	80,679	105	68

OTU clustering analysis was performed on cigar tobacco at a 97% similarity threshold, with the results presented in Table 1. The analysis revealed a total of 1,435 bacterial OTUs and 333 fungal OTUs, indicating a higher number of bacterial OTUs compared to fungal ones. The number of bacterial and fungal OTUs generally showed an increasing and then decreasing trend with fermentation at the three different water content conditions (Table 1).

**Figure 1:**
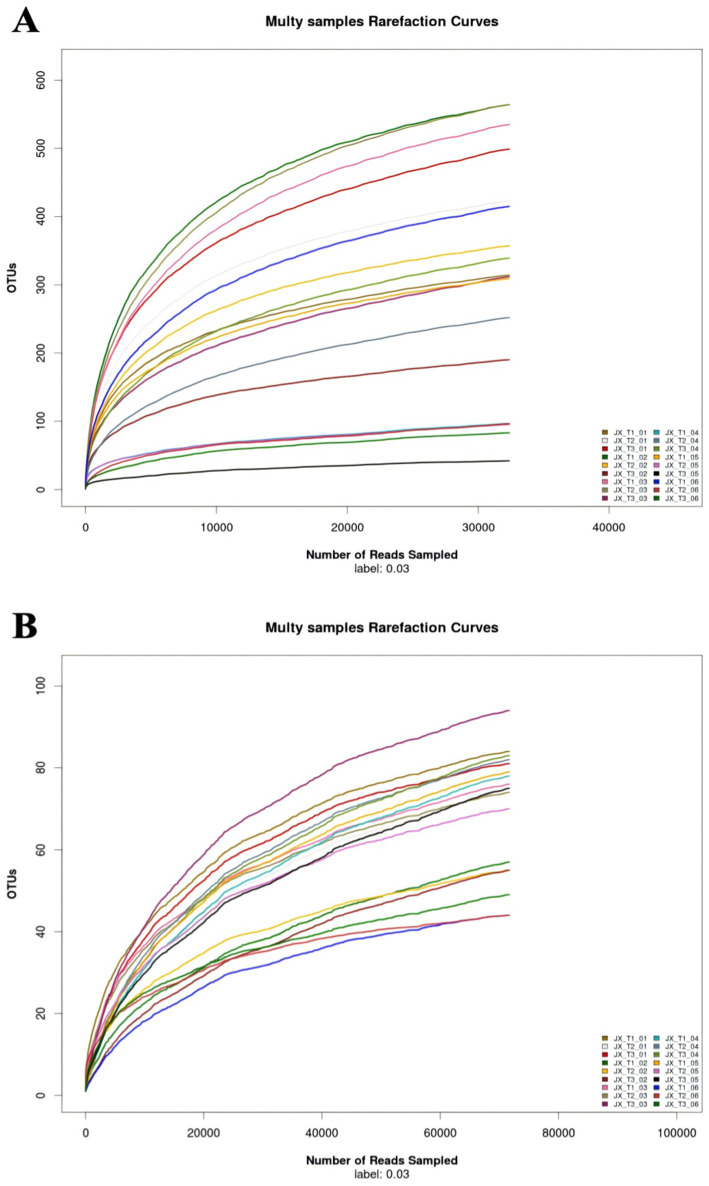
Dilution curves of bacteria **(A)** and fungi **(B)** in cigar filler leaves samples.

Dilution curves for both fungi and bacteria were generated from the sequencing data for each sample. For bacteria, the slopes of the curves initially increased, and once the sequencing count reached 15,000, all 18 dilution curves leveled off, suggesting that sequencing had reached saturation and was adequate to represent the microbial diversity in the samples (Figure 1A). In contrast, for fungi, the OTUs for each sample showed a gradual increase when the sequencing count exceeded 60,000 (Figure 1B). This suggests that the microbial diversity of cigar filler leaves remains stable with increased sequencing, although new genera may still be identified with deeper and more extensive sequencing.

### 3.2 Principal component analysis (PCA)

**Figure 2:**
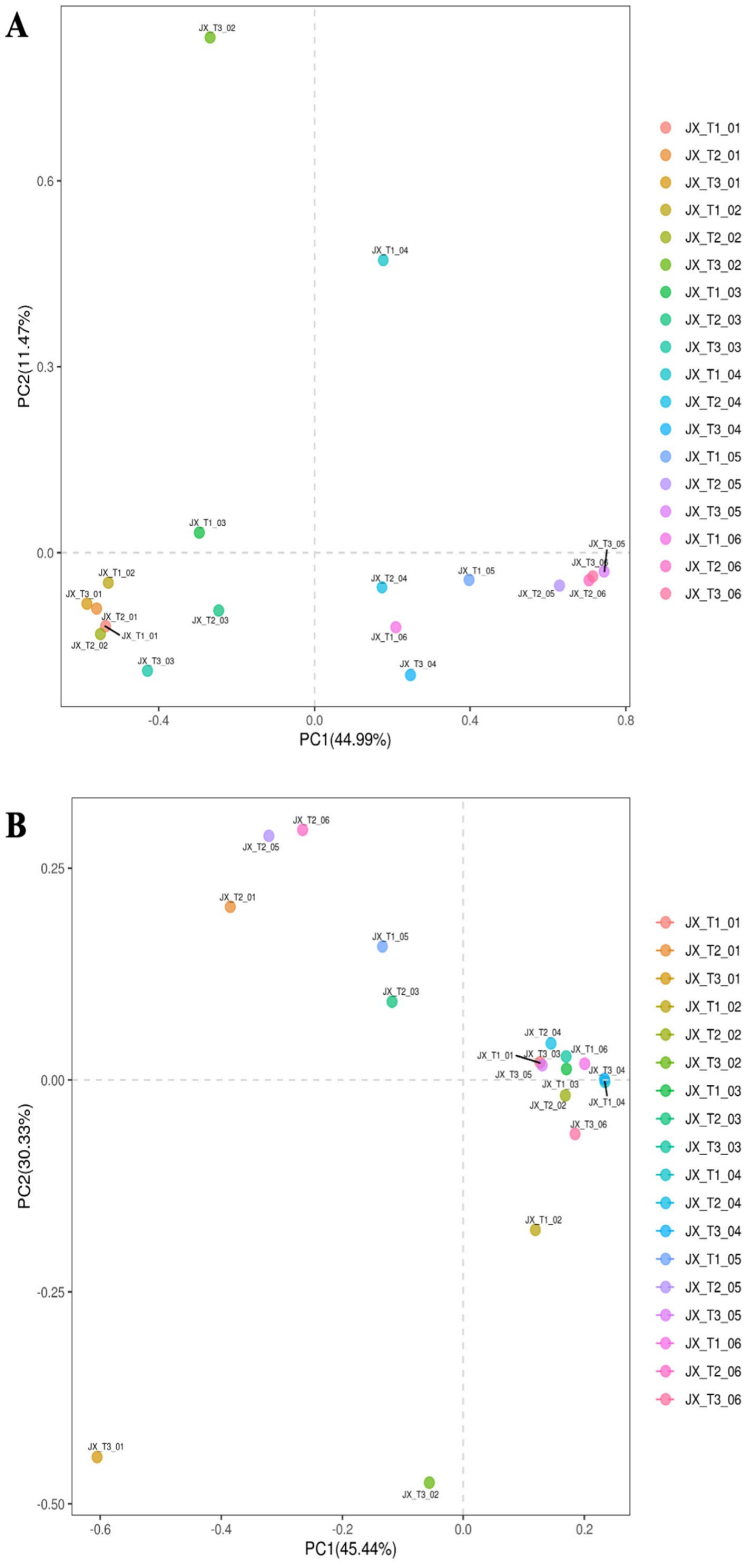
PCA of bacterial **(A)** and fungal **(B)** communities in cigar tobacco fermentation based on OTU level under different initial water content conditions.

The results of the principal component analysis (PCA) of the bacterial and fungal communities of different samples from cigar tobacco leaves fermented at three different initial water contents are shown in Figures 2A,B. Under the same initial water content, the samples at different fermentation stages were more dispersed, while those at the same fermentation stage were more dispersed at different initial water contents, but some samples were clustered together. This indicates that the microbial communities of cigar tobacco leaves with different initial water contents are changing and differing under artificial fermentation conditions, which suggests that the three initial water content treatments are comparable.

### 3.3 Venn diagram

**Figure 3:**
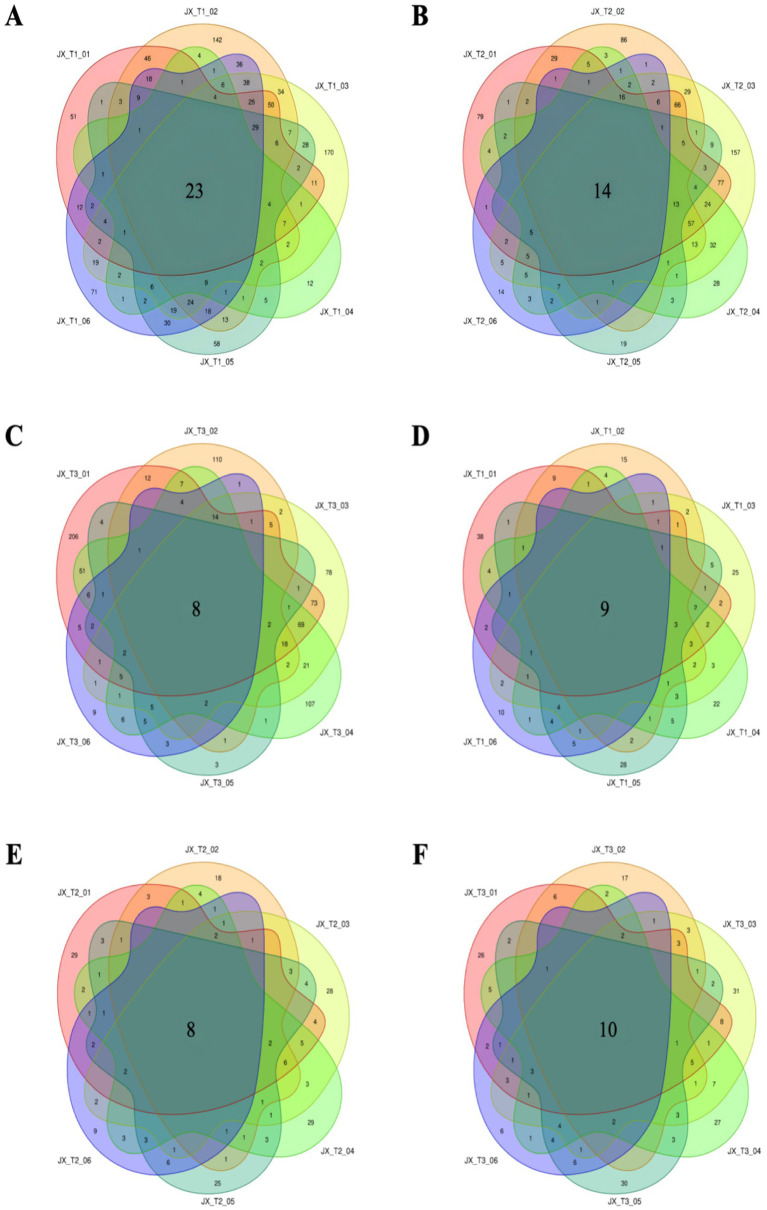
Number of common and unique OTUs of bacteria **(A–C)** and fungi **(D–F)** during the fermentation of cigar tobacco under different initial water contents.

At an initial water level of 20%, the unique bacterial and fungal OTUs in tobacco were 51 and 38, respectively, prior to fermentation. The number of unique bacterial OTUs in tobacco exhibited a pattern of increase followed by decrease as fermentation time progressed (Figure 3A). During fermentation at 22% water content, there were 14 shared bacterial OTUs among the bacteria in cigar filler leaves. When the tobacco leaves were unfermented, the unique OTUs counted 79, and similar to the 20% moisture scenario, the number of unique OTUs fluctuated, increasing initially and then decreasing with longer fermentation times (Figure 3B). At an initial water content of 24%, the unique OTUs in the tobacco leaves also started at 79, but gradually declined as fermentation continued, reaching a minimum during the late fermentation stage. Overall, the unique OTUs in tobacco decreased with extended fermentation, hitting the lowest point in the later stages. This suggests significant variations in bacterial OTUs under different initial water conditions, indicating substantial changes in the bacterial community (Figure 3C). Across the three moisture conditions, the number of fungal-specific OTUs generally decreased throughout the fermentation process, with minor fluctuations (Figures 3D–F). These findings suggest that a diverse range of microbial species was present on the surface of tobacco leaves before fermentation, which diminished post-fermentation, aligning with previous research (Liu et al., 2021).

### 3.4 Analysis of alpha diversity of microorganisms in cigar filler leaves with varying initial water contents

Alpha diversity indices, such as Shannon, Chao1, and Simpson, are commonly used to express the diversity of microbial community species, offering a thorough assessment of the richness of species composition within a community. The microbial Coverage index of all samples was 1.0, indicating that the sequences in the sample libraries were basically sequenced, i.e., the sample sequencing results could reflect the real situation of the samples. At a 97% similarity threshold, the Chao1 index for bacteria ranged from 72.802 to 860, the Shannon index from 0.8 to 5.88, and the Simpson index from 0.21 to 0.96. For fungi, the Chao1 index ranged from 72.25 to 204.5, the Shannon index from 0.28 to 2.42, and the Simpson index from 0.06 to 0.73 (Table 2).

**Table 2:** Microbial alpha diversity analysis in the fermentation process of cigar filler leaves under different water content conditions.

Sample	Fermentation stage	Initial water content (%)	Coverage index	Shannon		Chao1		Simpson	
				Bacteria	Fungi	Bacteria	Fungi	Bacteria	Fungi
JX-T1-01	Initial stage	20	1.00	4.34	0.55	376.12	95.67	0.12	0.88
JX-T1-02			1.00	5.45	0.36	609.52	90.33	0.07	0.89
JX-T1-03	Middle stage		1.00	5.56	0.34	602.32	101.09	0.06	0.93
JX-T1-04			1.00	3.64	0.12	130.21	105.07	0.10	0.98
JX-T1-05	Late stage		1.00	4.10	0.79	371.57	100.67	0.20	0.67
JX-T1-06			1.00	4.07	0.06	279.91	49.50	0.25	0.99
JX-T2-01	Initial stage	22	1.00	5.03	1.42	488.54	124.06	0.08	0.43
JX-T2-02			1.00	4.46	0.18	414.37	138.10	0.10	0.97
JX-T2-03	Middle stage		1.00	5.76	0.92	618.10	204.50	0.04	0.68
JX-T2-04			1.00	2.74	0.20	339.39	180.93	0.36	0.96
JX-T2-05	Late stage		1.00	1.81	1.75	126.23	125.06	0.57	0.40
JX-T2-06			1.00	0.99	1.69	281.25	94.67	0.76	0.45
JX-T3-01	Initial stage	24	1.00	4.88	1.65	588.46	134.53	0.13	0.36
JX-T3-02			1.00	4.00	0.92	224.14	87.00	0.11	0.57
JX-T3-03	Middle stage		1.00	4.66	0.20	444.55	185.12	0.09	0.96
JX-T3-04			1.00	3.95	0.11	417.71	153.75	0.18	0.98
JX-T3-05	Late stage		1.00	0.72	0.22	48.60	168.25	0.80	0.95
JX-T3-06			1.00	1.06	0.27	104.23	126.50	0.73	0.94

The results indicated that the Chao1 index for bacteria was higher than that for fungi, suggesting greater bacterial species richness in the tobacco samples. Throughout the fermentation of cigar filler leaves, the richness and diversity of the microbial community continuously evolved. Under three different initial water content conditions, the abundance of both bacterial and fungal communities initially increased and then decreased as fermentation progressed, peaking during the middle and late stages. The Shannon index for both bacteria and fungi exhibited a notable decline, while the Simpson index showed a significant increase, indicating that the diversity of bacterial and fungal communities was considerably higher during the early and middle stages of fermentation compared to the late stage, where diversity decreased.

### 3.5 Composition of bacterial communities

**Figure 4:**
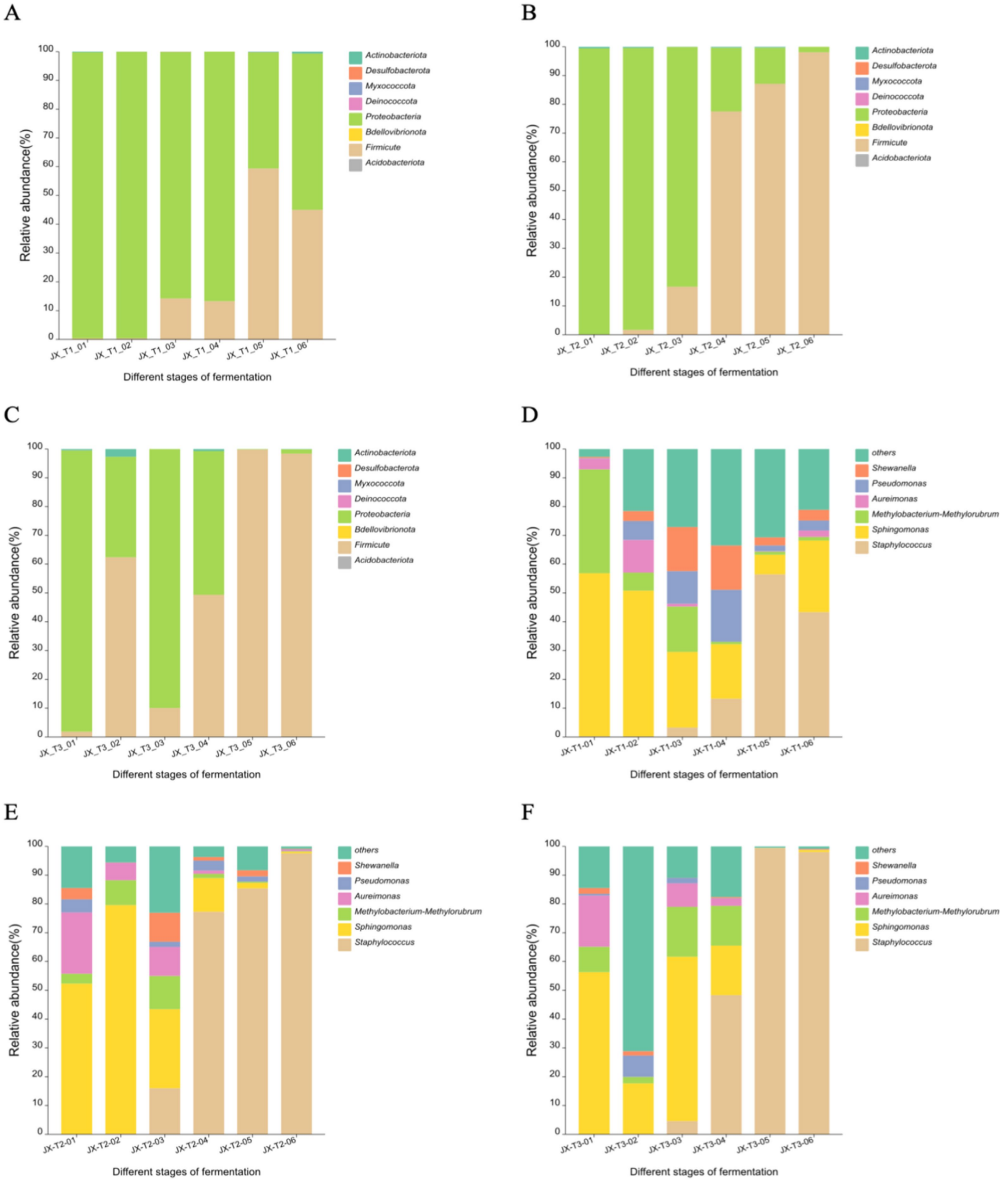
Bacterial community structure at the phylum level **(A-C)** and genus level **(D-F)** during the fermentation of cigar filler leaves under different water content conditions.

The figures illustrate that the three most abundant bacterial phyla on the surface of cigar filler leaves during fermentation were *Proteobacteria*, *Firmicutes*, and *Actinobacteria*, in that order. At an initial water content of 20%, *Firmicutes* and *Actinobacteria* were the most prevalent. The relative abundance of *Firmicutes* increased over time, rising from 0.25 to 59.32% after air curing, while *Proteobacteria* exhibited a decreasing trend, falling to 40.46% by the end of fermentation (Figure 4A). With an initial water content of 22%, the changes in *Proteobacteria* and *Firmicutes* were more pronounced. *Proteobacteria* were predominantly found in the early fermentation stages, with a relative abundance of 99.37% that significantly dropped to 1.87% in the middle and late stages, while *Firmicutes*’ relative abundance surged from 0.09 to 98.13% (Figure 4B). At an initial water content of 24%, *Actinobacteria* were present during the first and middle fermentation stages, with relative abundance ranging from 0.42 to 2.68%, before declining in the later stages. The dominant phylum shifted from *Proteobacteria* to *Firmicutes*, with the latter’s relative abundance increasing from 1.77 to 98.44% (Figure 4C).

At the genus level, the four most abundant genera were *Staphylococcus*, *Sphingomonas*, *Methylobacterium-Methylorubrum*, and *Pseudomonas*. With an initial water content of 20%, Staphylococcus spp. and *Pseudomonas* spp. initially increased and then decreased over fermentation time, with *Staphylococcus* spp. being the most abundant in the late fermentation stage at 56.45%, *Sphingomonas* spp. and *Methylobacterium-Methylorubrum* spp. generally decreased, with their proportions falling from 56.59 and 36.10% to 24.98 and 1.23%, respectively (Figure 4D). At an initial water content of 22%, *Staphylococcus* spp. showed a significant increase in the middle and late fermentation stages, rising from 1.85 to 97.61%. *Sphingomonas* spp. displayed a pattern of increase followed by a decrease, peaking at 79.45%. *Methylobacterium-Methylorubrum* spp. and *Aureimonas* spp. were primarily found in the early and middle stages, declining to below 1% in the later stages (Figure 4E). At 24% initial water content, *Staphylococcus* spp. significantly increased in the middle and late stages, reaching a relative abundance of 99.39%. *Sphingomonas* spp. and *Methylobacterium-Methylorubrum* spp. were mainly present in the early and middle stages, with their abundance dropping below 1% in the later stages (Figure 4F).

### 3.6 Composition of fungal communities

**Figure 5:**
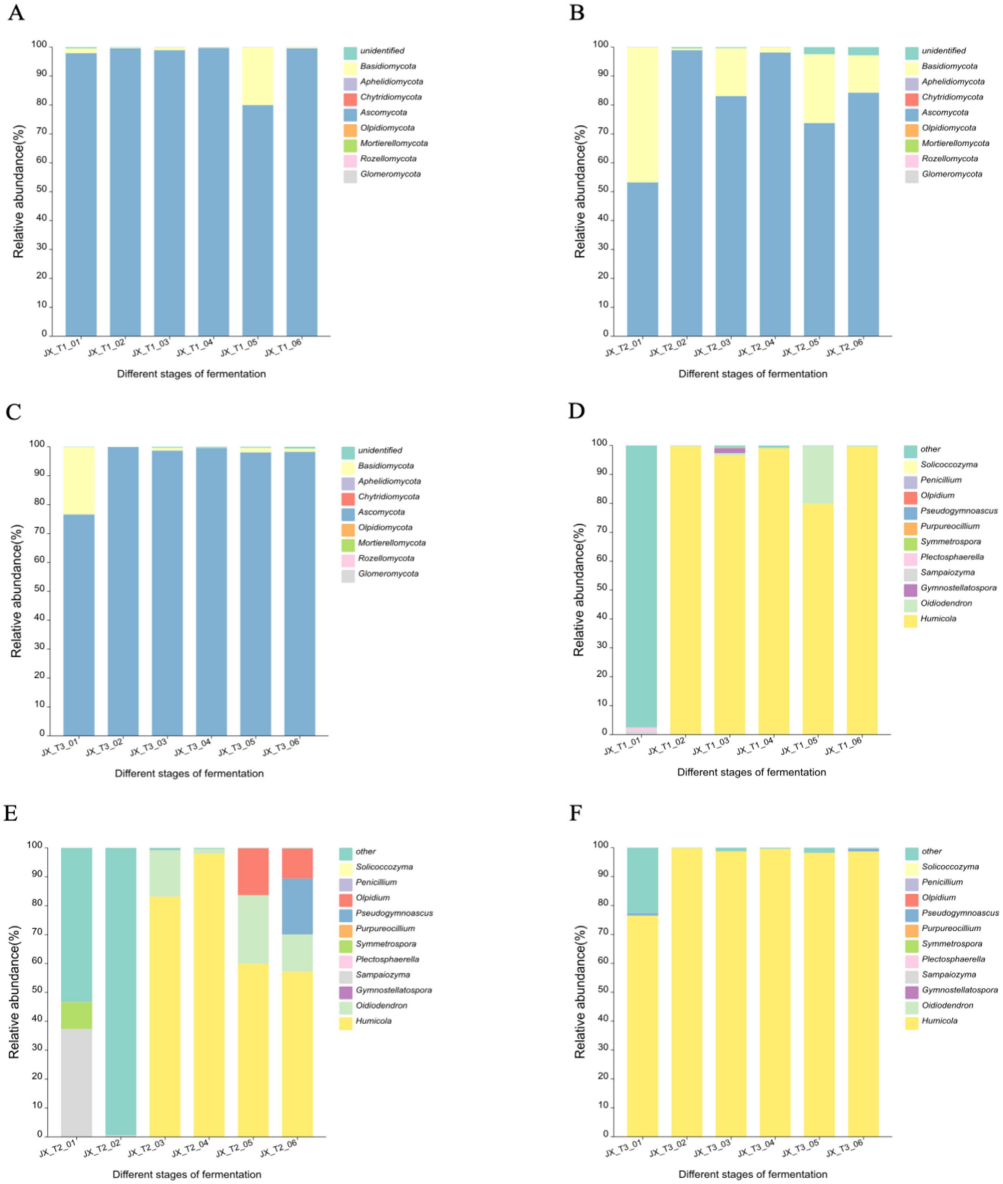
Community structure of fungi at phylum level **(A-C)** and genus level **(D-F)** during the fermentation of cigar filler leaves under different water content conditions.

The figures illustrate that the operational taxonomic units (OTUs) were classified into eight fungal phyla across six fermentation time stages and three different initial water content levels: *Ascomycota*, *Basidiomycota*, *Mortierellomycota*, *Chytridiomycota*, *Olpidiomycota*, *Rozellomycota*, *Glomeromycota*, and *Aphelidiomycota*. *Ascomycota* emerged as the predominant phylum, exhibiting a relative abundance of up to 99% across the three initial water content conditions. Additionally, *Basidiomycota* displayed notable fluctuations at 22 and 24% water content, showing a decline in abundance as fermentation continued, with over 15% abundance at the 22% water content level. The other fungal phyla did not exhibit significant variations during fermentation (Figures 5A–C).

At the genus level, the three most abundant fungal genera across the three initial water content conditions were *Humicola*, *Oidiodendron*, and *Pseudogymnoascus*. *Humicola* spp. was absolutely dominant in the fermentation of cigar tobacco. At 20 and 24% initial water content, *Humicola* spp. remained relatively stable in relative abundance throughout most of the fermentation (Figures 5D,F). The diversity of fungal genera was greater under the 22% water condition than under the other two water conditions. The relative abundance of *Sampaiozyma* spp. reached 37.32% at the very beginning of fermentation and then decreased to less than 1%; *Humicola* spp. and *Oidiodendron* spp. appeared in the middle and late stages of fermentation and the relative abundance decreased to 57.14 and 12.90%, respectively, as fermentation progressed; *Olpidium* spp. and *Pseudogymnoascus* spp. appeared in the later stages with relative abundances of 16.21 and 19.30%, respectively, (Figure 5E).

### 3.7 Analysis of symbiotic networks among key microorganisms on cigar filler leaves surfaces

**Figure 6:**
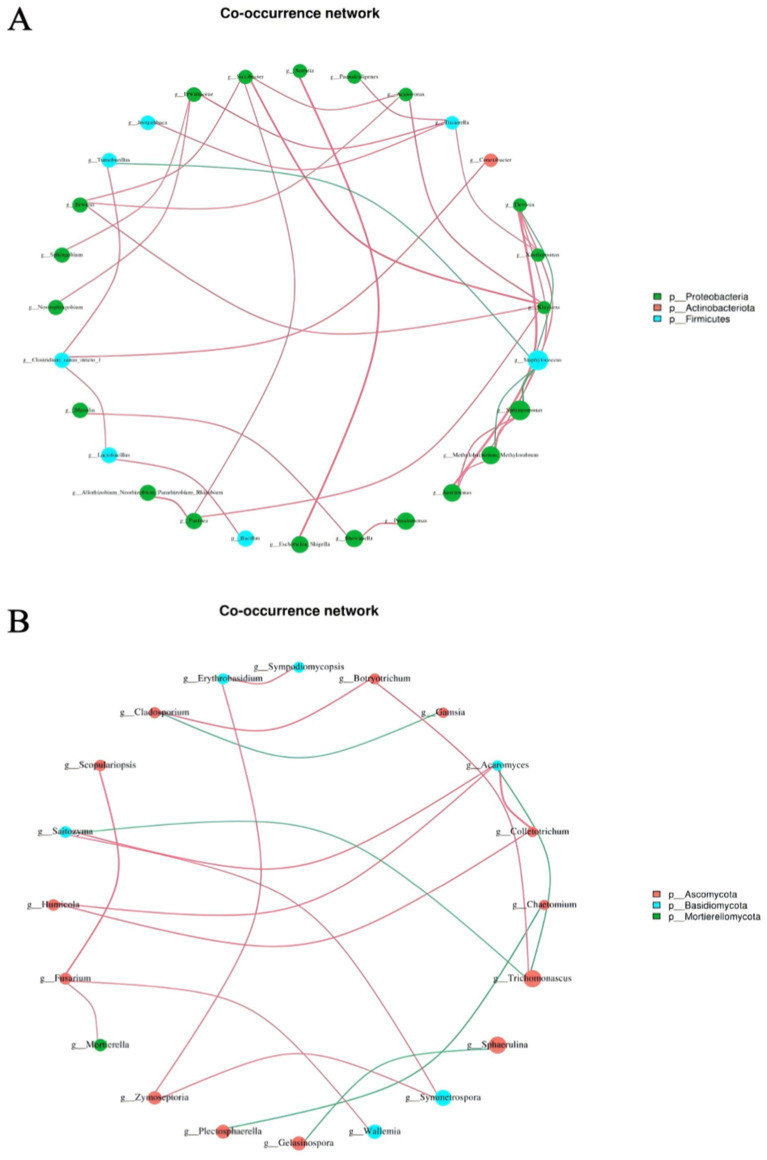
Correlation network analysis between bacterial **(A)** and fungal **(B)** genus. The node colors indicate their respective phyla, the size reflects their abundance, and the line thickness denotes the strength of correlation; the colors of the dots also represent their phyla, with a red line indicating a positive correlation and a green line indicating a negative correlation.

Microorganisms exhibit close interrelations (Hughes et al., 2008), biotic interactions are a major factor in shaping community structure. (Mould and Hogan, 2021) and the correlation network analysis among various microbial genera is illustrated in Figure 6. A total of 28 bacterial nodes and 20 fungal nodes showed significant correlations, highlighting the intricate relationships among different microorganisms. The five most abundant bacterial genera during the fermentation of cigar tobacco were *Staphylococcus* spp., *Sphingomonas* spp., *Methylobacterium-Methylorubrum* spp., *Pseudomonas* spp., and *Aureimonas* spp., which exhibited the most complex interactions with other genera, suggesting their crucial role in tobacco fermentation. *Staphylococcus* spp. displayed a negative correlation with *Sphingomonas* spp. and *Methylobacterium-Methylorubrum* spp., while showing a positive correlation with other predominantly central bacteria (Figure 6A). There must be a non-cooperative relationship among *Staphylococcus* spp., *Sphingomonas* spp. and *Methylobacterium-Methylorubrum* spp., such as competition, parasitism, predation, and antagonism. Their demise would bring about dramatic changes in community structure and functioning.

Among the dominant fungal genera, *Humicola* spp. demonstrated a positive correlation with *Colletotrichum* spp. and *Acaromyces* spp., suggesting a cooperative relationship among them as well (Figure 6B). The abundance and interactions of dominant microorganisms during the fermentation of cigar tobacco leaves influenced the changes in the characteristic microorganisms in the microbial community, which in turn influenced the changes in the microbial community structure in cigar tobacco leaves (Wu X. et al., 2023).

### 3.8 Predicting the functions of bacterial communities on cigar filler leaves

**Figure 7:**
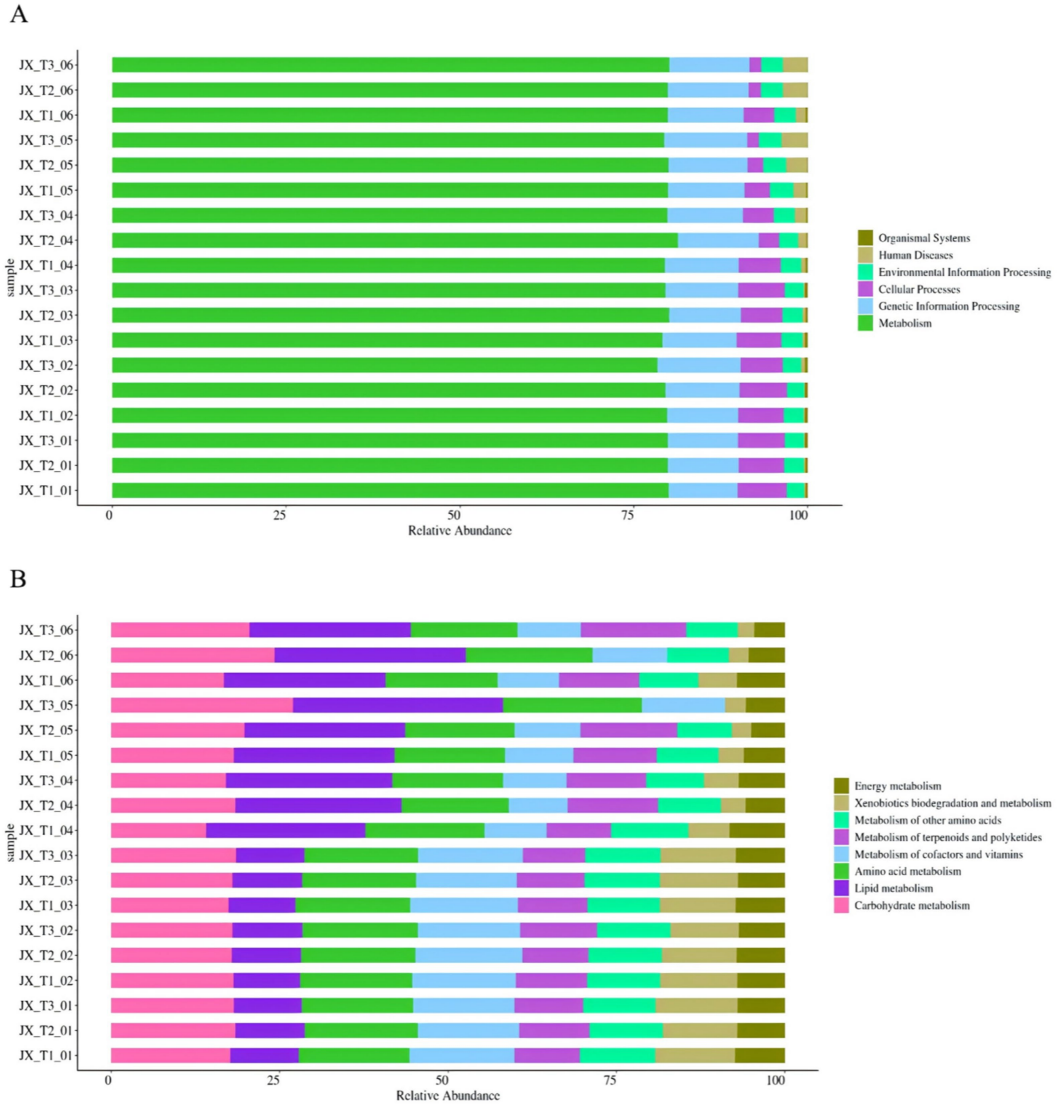
Primary functional analysis of predicted genes **(A)** Secondary functional analysis of predicted genes **(B)**.

The functional prediction of 16S amplicon sequencing data was conducted using the PICRUSt2 analysis platform, comparing the resulting OTU abundance table with the Kyoto Encyclopedia of Genes and Genomes (KEGG) database. This approach can initially highlight the functional differences in bacterial communities across various samples (Langille et al., 2013; Abia et al., 2018). The findings indicated that all samples exhibited similar KEGG pathway abundances, encompassing six primary biometabolic pathways: organismal systems, human diseases, environmental information processing, cellular processes, genetic information processing, and metabolism. Among these, metabolism emerged as the most prominent primary function (Figure 7A).

A more detailed analysis of the predicted genes at the secondary functional level revealed 36 secondary and 167 tertiary sub-functions. The figure illustrates the top 8 sub-functional layers along with their respective abundances in the secondary functional layer (Figure 7B). These activities are essential for the production of aroma precursors, which significantly enhance the smoking quality of cigarettes and improve the overall quality of tobacco (Weng et al., 2024).

## 4 Discussion

In this study, we performed 16S rRNA and ITS1 high-throughput sequencing of microorganisms in the fermentation process of cigar filler leaves under different initial water content conditions and revealed the changes in microbial community structure and function through Alpha diversity analysis and PICRUSt function prediction to study the changes in the microbial community in the cigar filler leaves under different initial water conditions, which is conducive to clarifying the role of microorganisms in fermentation.

### 4.1 Analysis of microbial diversity and community changes in cigar filler leaves

The results of Alpha diversity showed that the abundance and diversity of bacterial communities in the three initial water contents of cigar filler leaves varied considerably during the fermentation process, with the diversity and abundance of bacterial communities in the pre-fermentation tobacco being significantly higher than that in the post-fermentation tobacco. In contrast, the abundance and diversity of fungal communities varied less, but showed an overall trend of increasing and decreasing with the increase in fermentation time. At the same fermentation time, the abundance and diversity of the bacterial community were significantly higher than that of the fungal community, indicating that bacteria played a dominant role in the fermentation process of aubergine tobacco.

Significant variations were observed in the microbial community composition of cigar filler leaves prior to and following fermentation. The *α*-diversity of both bacteria and fungi exhibited a decline during the later stages of fermentation, likely due to certain microorganisms being unable to thrive under fermentation conditions, leading to their growth being suppressed throughout the process (Huang et al., 2010).

At the bacterial phylum level, the most prevalent phyla during fermentation under varying initial water content conditions were *Proteobacteria*, *Firmicutes*, and *Actinobacteria*. This aligns with findings from a previous study on cigar tobacco leaves (Zhang et al., 2023). These three phyla of microorganisms are also widely distributed in the soil between plant roots and play an important role in plant life (Kinkel et al., 2011).

At the bacterial genus level, the primary genera within the bacterial community—*Sphingomonas* and *Methylobacterium-Methylorubrum* were mainly present in the first and middle stages of cigar tobacco leaves fermentation. Their numbers gradually decreased in the later stages of fermentation, which may be due to the increase in fermentation temperature and the inability to adapt to the growth of the microbial community. The changes in the microbial community were mainly related to their different functions during fermentation, which showed that they played a more prominent role in the pre-fermentation period. *Pseudomonas* spp. and *Aureimonas* spp. initially increased and then decreased over fermentation time, which may be because the first and middle stages of fermentation are more suitable for its growth, as influenced by the tobacco leaves’ chemical composition, the tobacco’s endophytes, and the fermentation environment. This observation is consistent with findings from a study that analyzed the diversity of bacterial communities in H382 cigar tobacco across different fermentation periods using 16sRNA sequencing (Zhang et al., 2021). At 22 and 24% water content conditions, *Aureimonas* spp. was predominantly present in the pre-fermentation phase, possibly due to its ability to adapt to higher water content conditions. *Staphylococcus* spp. was dominant in the late stage of fermentation under the three different initial water content conditions, which may be due to the decrease in tobacco moisture content and increase in pH during fermentation, and the strain’s suitable alkali and salt tolerance, which made it dominant in stack fermentation (Di Giacomo et al., 2007). However, the 20% water content condition showed a decrease in the number of *Staphylococcus* spp. at the end of fermentation, possibly due to chance, this did not occur in the other two water content conditions. It has now been shown that *Staphylococcus* spp. can rapidly metabolize malic and citric acids in tobacco and that these organic acids affect tobacco smoking quality (Di Giacomo et al., 2007). Furthermore, it is known to participate in sugar and fat metabolism in food, producing aromatic compounds like aldehydes and methyl ketones, making it a key genus in flavor development (Sondergaard and Stahnke, 2002; Schlegel et al., 2021). Functional predictions indicate that *Pseudomonas* can break down various environmental pollutants, with some strains capable of degrading harmful substances in tobacco. There is a growing body of research on its role in nicotine degradation (Ruan et al., 2005), as well as its contribution to adjusting tobacco strength and enhancing the quality of upper tobacco (Zhong et al., 2010; Zhao et al., 2012), for instance, *Pseudomonas_vulpinum* was positively correlated with Nonanal and favored the accumulation of citrus-like aromas (Wu X. et al., 2023). Fermentation enhances the flavor of cigar tobacco leaves, and *Sphingomonas* has gained attention in recent years for its extensive metabolism of aromatic compounds, with metabolites like phytosphingosine and sphingomyelin being vital in tobacco fermentation (Li et al., 2020). *Sphingomonas* produce aldehydes, esters, and ketones during reproductive metabolism (Hu et al., 2019).

In the fungal phylum classification, *Ascomycota* and *Basidiomycota* were the most prevalent, aligning with findings from a study on the dominant fungal flora in cigar tobacco using high-throughput sequencing (Zhang et al., 2023). They are highly adaptable and can survive and reproduce in different environmental conditions, which may be one of the reasons why they are a dominant phylum. The efficient utilization of nutrients leads to their higher abundance (Chen et al., 2017).

At the genus level, *Humicola* was the most dominant, which contrasts with previous studies. This discrepancy may arise from various factors influencing the fungal community structure during fermentation, including the initial water content of the cigar tobacco leaves, the characteristics of the leaves themselves, and fluctuations in moisture during the fermentation process. Additionally, some fungi present in the tobacco leaves may have struggled to adapt to the artificial fermentation conditions, leading to inhibited growth. The relative abundance of *Humicola* decreased during the later stages of fermentation at initial moisture levels of 20 and 22%, potentially due to pH changes within the tobacco leaves during fermentation (Wu et al., 2022; Zhao et al., 2024). Generally, *Humicola* spp. is commonly found in soils rich in organic matter, and the heat-stable enzymes and metabolites it produces, such as cellulase, hemicellulase, ligninase, amylase, and glucose isomerase, have significant practical applications (Yang et al., 2015). This fungus is essential for the fermentation process and the production of solid brewer’s yeasts, suggesting it plays a crucial role in breaking down macromolecules during tobacco fermentation (Liu et al., 2021; Su et al., 2016). The composition of fungal genera at 22% initial water content was more different from the other two conditions, possibly due to the samples’ variability. The relative abundance of *Sampaiozyma* reached 37.32% during the pre-fermentation period, which is consistent with previous studies that detected the presence of *Sampaiozyma* spp. in cigar tobacco leaves (Wang et al., 2024); at the end of fermentation, *Pseudogymnoascus* was detected and has been shown. Fungi of the genus *Pseudogymnoascus* promote crop biomass and assist in resistance to pathogenic fungi in continuous soil environments (Liu et al., 2022). The appearance of *Olpidium* spp. at the end of fermentation may be due to its growth being inhibited by the competitive relationship between the pre-fungal communities. Less has been reported about it in tobacco. However, it is a dominant fungal genus in the inter-root soil microorganisms of wheat, oilseed rape, and tomato, and its relative abundance was positively correlated with fast-acting phosphorus content (Baruah et al., 2019; Hilton et al., 2013). Its specific role in the fermentation of cigar tobacco leaves requires further research and investigation. *Oidiodendron* spp. has been less studied in tobacco, but it is the fungus most isolated from rhododendron family roots and most easily cultivated and formed into rhododendron-like mycorrhizal structures (Kerley and Read, 1997). These fungi can secrete cell wall degrading enzymes and proteases to break down organic matter into small nitrogen molecules and enhance the plant body’s acquisition of inorganic nitrogen and phosphorus. In addition, *Oidiodendron* spp. produces microbial extracellular polysaccharides that have been shown to have good solubility (Govindarajulu et al., 2005).

At the same time, utilizing the symbiotic network to study the relationship between the dominant genera can lay a theoretical foundation for the later stage to significantly promote the growth of some functional microorganisms through the exogenous addition of microorganisms (Zheng et al., 2022).

### 4.2 Prediction of bacterial function in cigar tobacco fermentation

Research has highlighted the crucial role of microbial communities and their activities in enhancing tobacco quality. The growth and metabolic processes of microorganisms lead to the breakdown or transformation of biomolecules, such as proteins, in tobacco, resulting in the production of various volatile aroma compounds and the reduction of impurities, thereby improving the quality of fermented tobacco (Wu et al., 2023; Zong et al., 2023). For instance, *Bacillus* produces a range of enzymes that decompose organic matter, contributing to the sweetness and smoothness of tobacco while minimizing irritation (Araujo et al., 2021). Functional predictions of the bacterial community in the samples indicated that over 75% of the metabolism was attributed to these microorganisms, suggesting that those on the surface of cigar filler leaves primarily participated in the fermentation process through metabolic pathways. The main pathways utilized by bacteria for macromolecule degradation included carbohydrate metabolism, lipid metabolism, and amino acid metabolism, aligning with previous findings (Weng et al., 2024). Intermediates from carbohydrate metabolic pathways can serve as raw materials for synthesizing various substances. Amino acids can engage in a Maillard reaction with reducing sugars, a key reaction responsible for aroma development in tobacco (Geng et al., 2024). The high relative abundance of energy metabolism indicates that it can supply energy for subsequent tobacco fermentation (Yan et al., 2022).

### 4.3 Future direction

The study of microbial community changes during the fermentation of cigar tobacco leaves under different initial water content conditions is conducive to a better understanding of the dynamics of the dominant microorganisms and their role in fermentation. The organisms studied in the article contributed to the aroma of cigar tobacco leaves, a reference for establishing the correlation analysis between dominant microorganisms and flavor substances and producing cigar products with characteristic aromas. PICRUSt function prediction has limitations; functional genes of microorganisms can be further explored by macro-genome sequencing to improve the fermentation efficiency of cigar tobacco leaves. In addition, the article studied cigar tobacco leaves from Anhui Province as a representative, after which the sampling area can be further expanded to make the analysis more generalized. In generalized research, a complete system of test methods can be developed to control the test conditions, thus improving reproducibility strictly.

## 5 Conclusion

This study examines the variations in microbial community diversity, structural composition, and bacterial community function predictions during the fermentation of cigar filler leaves under different initial water content conditions. The initial water content significantly influenced the composition and relative abundance of microbial communities during fermentation, resulting in similar community compositions but differing abundances. Bacterial diversity was found to be greater than that of fungi throughout the fermentation process, with an overall decline in both bacterial and fungal diversity and abundance as fermentation time increased. The dominant bacterial phyla included *Proteobacteria*, *Firmicutes*, and *Actinobacteria*, while the predominant fungal phyla were *Ascomycota* and *Basidiomycota*. The leading bacterial genera were *Staphylococcus*, *Sphingomonas*, *Methylobacterium*—*Methylorubrum*, and *Pseudomonas*, which exhibited an initial increase followed by a decrease. The main fungal genus identified was *Humicola*. The functional predictions for bacteria were primarily focused on carbohydrate metabolism, lipid metabolism, and amino acid metabolism. This research provides a scientific foundation for further understanding the microbial fermentation mechanisms of cigar tobacco leaves and identifying the dominant functional bacteria.

## Data Availability

Original datasets are available in a publicly accessible repository: The original contributions presented in the study are publicly available. This data can be found here: https://www.ncbi.nlm.nih.gov/sra/PRJNA1211815.
